# Flexible and monolithically integrated multicolor light emitting diodes using morphology-controlled GaN microstructures grown on graphene films

**DOI:** 10.1038/s41598-020-76476-6

**Published:** 2020-11-12

**Authors:** Keundong Lee, Dongha Yoo, Hongseok Oh, Gyu-Chul Yi

**Affiliations:** Department of Physics and Astronomy, Institute of Applied Physics, and Research Institute of Advanced Materials (RIAM), Seoul National University, Seoul, 151-747 Korea

**Keywords:** Nanoscience and technology, Lasers, LEDs and light sources

## Abstract

We report flexible and monolithically integrated multicolor light-emitting diode (LED) arrays using morphology-controlled growth of GaN microstructures on chemical-vapor-deposited (CVD) graphene films. As the morphology-controlled growth template of GaN microstructures, we used position-controlled ZnO nanostructure arrays with different spacings grown on graphene substrates. In particular, we investigated the effect of the growth parameters, including micropattern spacings and growth time and temperature, on the morphology of the GaN microstructures when they were coated on ZnO nanostructures on graphene substrates. By optimizing the growth parameters, both GaN microrods and micropyramids formed simultaneously on the graphene substrates. Subsequent depositions of InGaN/GaN quantum well and *p*-GaN layers and *n*- and *p*-type metallization yielded monolithic integration of microstructural LED arrays on the same substrate, which enabled multicolor emission depending on the shape of the microstructures. Furthermore, the CVD graphene substrates beneath the microstructure LEDs facilitated transfer of the LED arrays onto any foreign substrate. In this study, Cu foil was used for flexible LEDs. The flexible devices exhibited stable electroluminescence, even under severe bending conditions. Cyclic bending tests demonstrated the excellent mechanical stability and reliability of the devices.

## Introduction

Highly efficient and flexible multicolor micrometer-scale light-emitting diodes (micro-LEDs) are required for mobile and wearable optoelectronic devices and displays with high resolution and low power consumption^1,2^. Organic materials have long been studied for such applications, but higher device performance is expected when using inorganic materials due to their excellent electrical and optical characteristics as well as long-term stability and reliability^3,4^. Notably, GaN based micro-LEDs are promising candidates due to their excellent brightness and long life. However, integration of micro-LEDs for full-color displays remains to be resolved. Previous integration methods using multiple transfer steps (pick-up and drop-off) onto a flexible substrate and self-assembly limit inorganic LEDs to high-resolution displays and are neither reliable nor reproducible for mass production. The integration issue would be resolved using monolithically integrated micro-LEDs to demonstrate multicolor emissions via tuning their bandgap by varying the In composition of the InGaN/GaN quantum well (QW)^5–7^. Meanwhile, recent studies on GaN based micro- and nanostructures on chemical-vapor-deposited (CVD) graphene films have demonstrated their suitability for flexible light-emitting-device applications^8,9^. Accordingly, monolithic integration of various GaN microstructures on CVD graphene films is a critical step for the fabrication of flexible and high-resolution micro-LED displays^10,11^. Herein, we report the simultaneous growth of GaN microrods and micropyramids on CVD graphene films and the fabrication of flexible monolithically integrated multicolor micro-LEDs using the microstructures.

## Materials and methods

### Material growth

The basic strategy for the growth of GaN layers on ZnO-nanostructured graphene films was as follows. The graphene thin films were synthesized on Cu foil and transferred onto SiO_2_/Si substrates. For selective growth of inorganic materials on a CVD graphene film, a 300-nm-thick SiO_2_ layer was deposited on the CVD graphene film and patterned by conventional lithography. Then, ZnO nanotubes were selectively grown on the CVD graphene film by catalyst-free MOVPE using high-purity diethylzinc and oxygen (O_2_) as the reactants for Zn and O, respectively. Herein, we prepared ZnO nanostructures with different spacings of 2, 4, 6, and 8 µm and a fixed height and diameter of 3.8 and 0.6 µm, respectively (Fig. S1). The growth of position-controlled ZnO nanotubes is detailed in our previous report^12^. Immediately following the ZnO nanostructure array preparation, *n*-GaN layers were grown along the circumferences of the nanostructure array using MOVPE^13^ for growth times of 2, 4, 10, 16, and 20 min. Heteroepitaxy of *n*-GaN on ZnO nanotubes was performed using a two-step growth method. For the first step, GaN was coated on the ZnO nanotubes using high-purity trimethylgallium and ammonia as the reactants for Ga and N under an N_2_ atmosphere at about 500 °C to prevent damage to the ZnO nanotubes. Subsequently, an additional *n*-GaN layer was deposited at a high temperature of about 1000 °C in an H_2_ atmosphere using di-*t*-butylsilane as the doping source. After *n*-GaN/ZnO heterostructure growth, In_*x*_Ga_1−*x*_N/GaN single QWs were grown at 760 °C and 850 °C, respectively. Finally, an Mg-doped *p*-GaN overlayer was deposited at about 1000 °C using bis(cyclopentadienyl)magnesium as the doping source^10^. The typical resistivity and carrier concentration of the *n*-GaN were measured to be 6 × 10^–3^ Ω cm and 4 × 10^18^ electrons cm^−3^, respectively; those of the *p*-GaN were 5–10 Ω cm and 0.8–1.3 × 10^17^ holes cm^−3^ after *p*-type activation at 700 °C.

### LED fabrication and transfer

To fabricate the LED devices, metal electrodes were made on the GaN microstructures by evaporating metals on a patterned area. Before depositing the metal thin films, a PI layer was spin-coated on the microstructures to support the metal electrodes. Then, after exposure of the *p*-GaN overlayer by PI etching using O_2_ plasma treatment, Au/Ni layers (20/20 nm) were deposited as *p*-contact electrodes of the micro-LEDs. Ohmic contact was achieved by rapid thermal annealing in ambient air at 300 °C for 5 min. Subsequently, the entire device structures fabricated on the CVD graphene films were released from the SiO_2_/Si substrate by selective etching of the SiO_2_ interlayer using buffered oxide etchant. The LEDs on the CVD graphene films were transferred onto plastic and Cu foil.

### Surface morphology characterization

The surface morphology of the ZnO nanostructures, GaN microstructures, and the monolithically integrated micro-LEDs was investigated using field-emission scanning electron microscopy (SUPRA55VP; Carl Zeiss).

### Electrical and EL characterization

The *I*–*V* characteristics and EL spectra of the devices were measured while applying a DC voltage to the device using a Keithley 2400 source meter. The EL spectra were measured using a monochromator (DM 150i; Dongwoo Optron) and a detection system equipped with a charge-coupled device (DU401A; Andor Technology).

## Results and discussion

### Morphology controlled growth of GaN microstructures and their monolithic integrations

**Figure 1:**
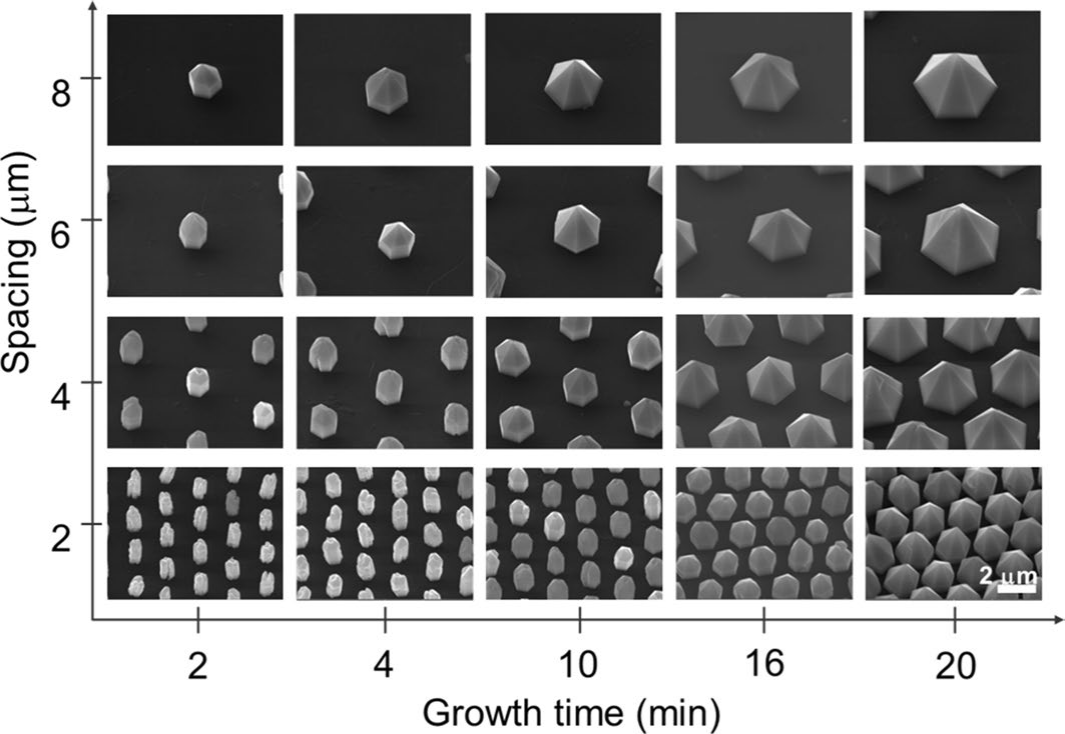
Morphology-controlled growth of GaN microstructures. The scanning electron microscope images of the GaN microstructures show that their surface morphology strongly depended on the growth time and the spacing of the ZnO nanotube array that was used as a growth template.

Simultaneous growth of different GaN microstructures was achieved by heteroepitaxial growth of GaN layers on position-controlled ZnO nanotube arrays with different spacings prepared on graphene films. For the graphene substrate preparation, graphene thin films were synthesized on Cu foil using conventional CVD, lifted-off by wet-chemical etching, and transferred onto SiO_2_/Si substrates. Using graphene films as the substrate for the GaN microstructure growth was essential to prepare high-quality GaN microstructures and to facilitate transfer of the microstructures to arbitrary substrates for flexible device applications^8,14,15^. For position-controlled growth of ZnO nanotubes on the graphene films, a 300-nm-thick SiO_2_ layer was deposited on the CVD graphene films and patterned to 0.6-μm-diameter hole arrays with different spacings of 2, 4, 6 and 8 μm by conventional lithography. Then, ZnO nanotubes were selectively grown on the graphene films and GaN layers were coated on the ZnO nanotubes by metal–organic vapor-phase epitaxy (MOVPE)^12^. For simultaneous and morphology-controlled growth of GaN microstructures, we investigated the effect of the GaN growth time and the spacing between ZnO nanotubes in their arrays on the morphology of GaN microstructures when GaN was coated on the ZnO nanostructures prepared on graphene. In this study, we prepared ZnO nanostructures with different spacings of 2, 4, 6, and 8 μm and a fixed height and diameter of 3.8 and 0.6 μm, respectively (Supplementary Fig. S1). Immediately following the growth of the ZnO nanostructure array, *n*-GaN layers were grown along the circumferences of the nanostructure array using MOVPE^13^ for various growth time of 2, 4, 10, 16, and 20 min. Figure 1 presents scanning electron microscopy (SEM) images that show the surface morphology of the GaN microstructures grown for different growth times and ZnO nanotube spacings. Typically, the morphology of the GaN microstructures changed from microrods to micropyramids with increasing growth time and spacing. For example, GaN microstructures grown on ZnO nanotubes with a spacing of 2 µm exhibited a microrod shape with rough surface morphology after growing for 2 min, but the surfaces became smooth with increasing growth time to 10 min. Their diameter and height increased from 0.8 and 4.0 µm to 1.1 and 4.2 µm, respectively. However, upon further increasing the growth time to 20 min, the surface morphology of the GaN microstructures dramatically changed to micropyramidal with an increased diameter of 2.0 µm and negligible change in height. Similar morphology change behavior with growth time was observed for the larger spacings. Meanwhile, lateral growth of the micropyramids occurred on the semipolar rather than on the nonpolar planes, presumably resulting from difficulty in achieving growth on the nonpolar facet^16^. Since the dangling bond density of the nonpolar $\{1\bar{1}00\}$ facet (12.1 nm^–2^) is smaller than that of the semipolar $\{1\bar{1}01\}$ facet (16.0 nm^–2^), growth along the semipolar facet would be energetically more favorable, thus resulting in the formation of micropyramids.

For a given growth time, the morphology of the GaN microstructures also depended on the spacing of the ZnO nanotubes. For example, GaN microstructures grown on ZnO nanotubes for 10 min exhibited microrods with a spacing of 2 µm or micropyramids with 8-µm spacing. Other GaN microstructures grown for 2, 4, 16, and 20 min demonstrated similar morphology change behavior that depended on the spacing of the ZnO nanotubes. The dependence of the morphology change of the GaN microstructure on the spacing of the ZnO nanotubes and growth time was attributed to surface diffusion of adatoms in the growth rate model. For a surface diffusion length of Ga atoms of $\fancyscript{l}$, the area that Ga atoms can diffuse is ${\fancyscript{l}}^{2}\pi$. In this area, the amount of Ga atoms that can be grown on a single nanostructure would be $\sim {\fancyscript{l}}^{2}\pi /\fancyscript{n}$ when the number of ZnO nanostructures in the area is $\fancyscript{n}$ and their spacing is smaller than $\fancyscript{l}$. At a growth temperature of 1000 °C, $\fancyscript{l}$ is estimated to be 10 μm^17^, so the growth rate could be significantly affected by the ZnO nanostructure spacing when it is narrower than 10 μm. When the spacing was wider than 10 µm, smaller micropyramids with poor growth selectivity were obtained (not shown). Accordingly, for larger spacing that was less than the surface diffusion length of 10 μm, more adatoms reached each microstructure, which enhanced the microstructure growth rate. The effect of surface diffusion of Ga adatoms on the surface morphology of the GaN microstructures was also confirmed by our supplementary study on the change in the surface morphology of the GaN microstructure as a function of growth temperature (Supplementary Fig. S2).

### Device fabrication of the monolithically integrated flexible LEDs

**Figure 2:**
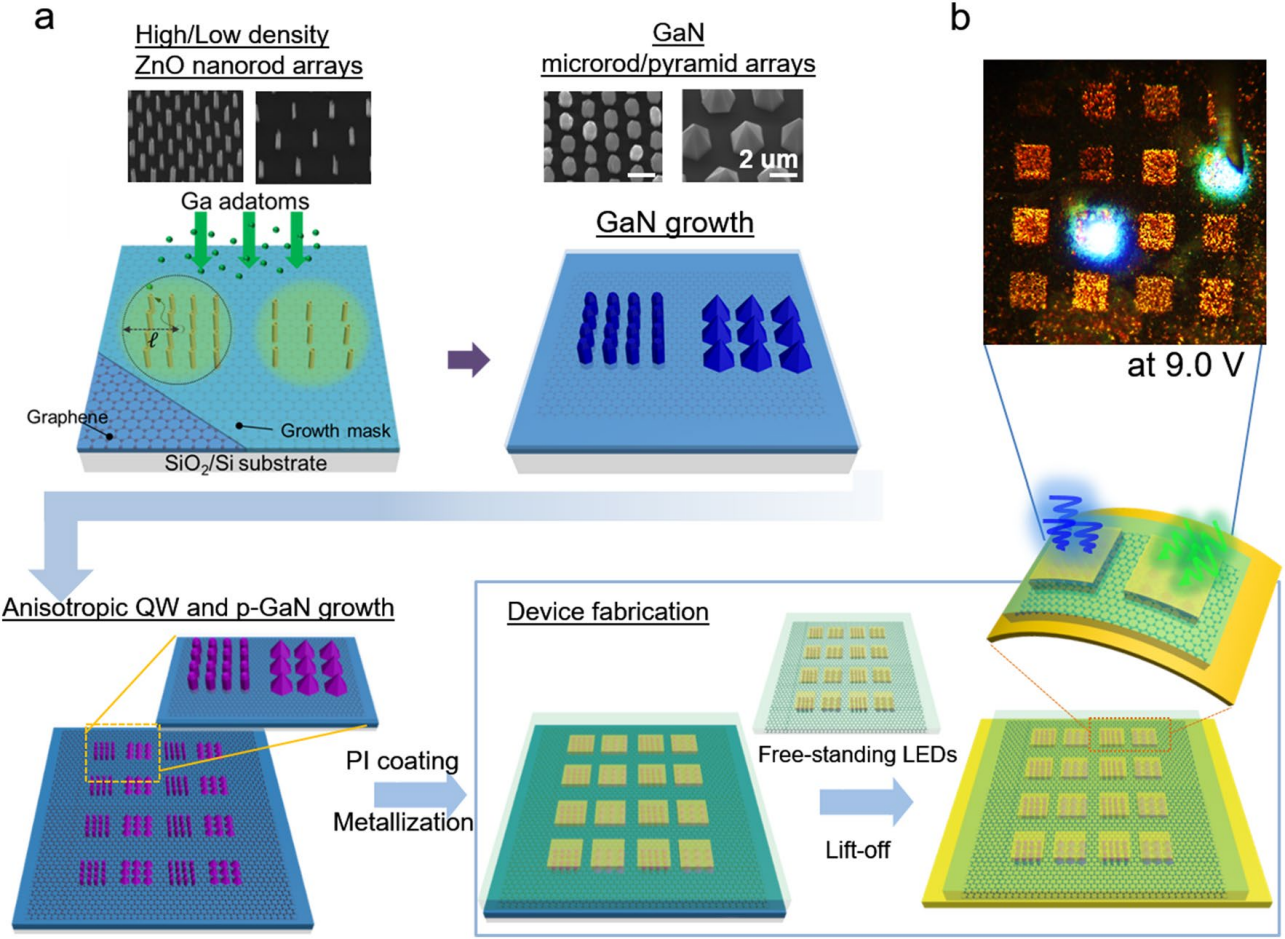
(**a**) Schematic illustration of the formation of the ZnO nanostructures template, monolithic integrated growth of GaN microrod and micropyramid arrays on CVD graphene film, and flexible light-emitting diode (LED) fabrication. (**b**) Photograph of light emission from the integrated micro-LEDs at an applied voltage of 9.0 V.

The morphology-controlled growth of GaN microstructures enabled the fabrication of monolithically integrated GaN microstructure LEDs that displayed multicolor light emission. The basic approach for the integrated micro-LED fabrication is shown in Fig. 2a. Both GaN microrod and micropyramid arrays were prepared on graphene films by simultaneously growing GaN layers on ZnO nanotubes with spacings of 2 and 6 µm for 10 min. Then, In_*x*_Ga_1−*x*_N/GaN QWs and *p-*GaN layers were heteroepitaxially grown on the GaN microstructures to form GaN *p–n* homojunction LEDs with QWs. After sequential steps of *p*-GaN activation, polyimide (PI) layer filling, and exposure of the PI layer on the *p*-contact area, integrated micro-LED arrays were fabricated by depositing Ni/Au metal contact layers with an area of 50 × 50 µm^2^ on the top region of the as-grown LEDs. For flexible device applications, the LEDs were removed from the SiO_2_/Si substrate by chemical etching of the SiO_2_ layer using buffered oxide etchant and transferred to Cu-coated polyethylene terephthalate films. The Cu layer was also used to make an electrical connection for flexible LEDs. Figure 2b presents optical microscope images showing the blue and green color emissions from the microrod- and micropyramid-based LED arrays, respectively, at the applied bias voltage of 9.0 V. Simultaneously applying the voltage to different multicolor LEDs enabled the fabrication of monolithically integrated multicolor LEDs.

### Device characteristics of monolithically integrated flexible LEDs

**Figure 3:**
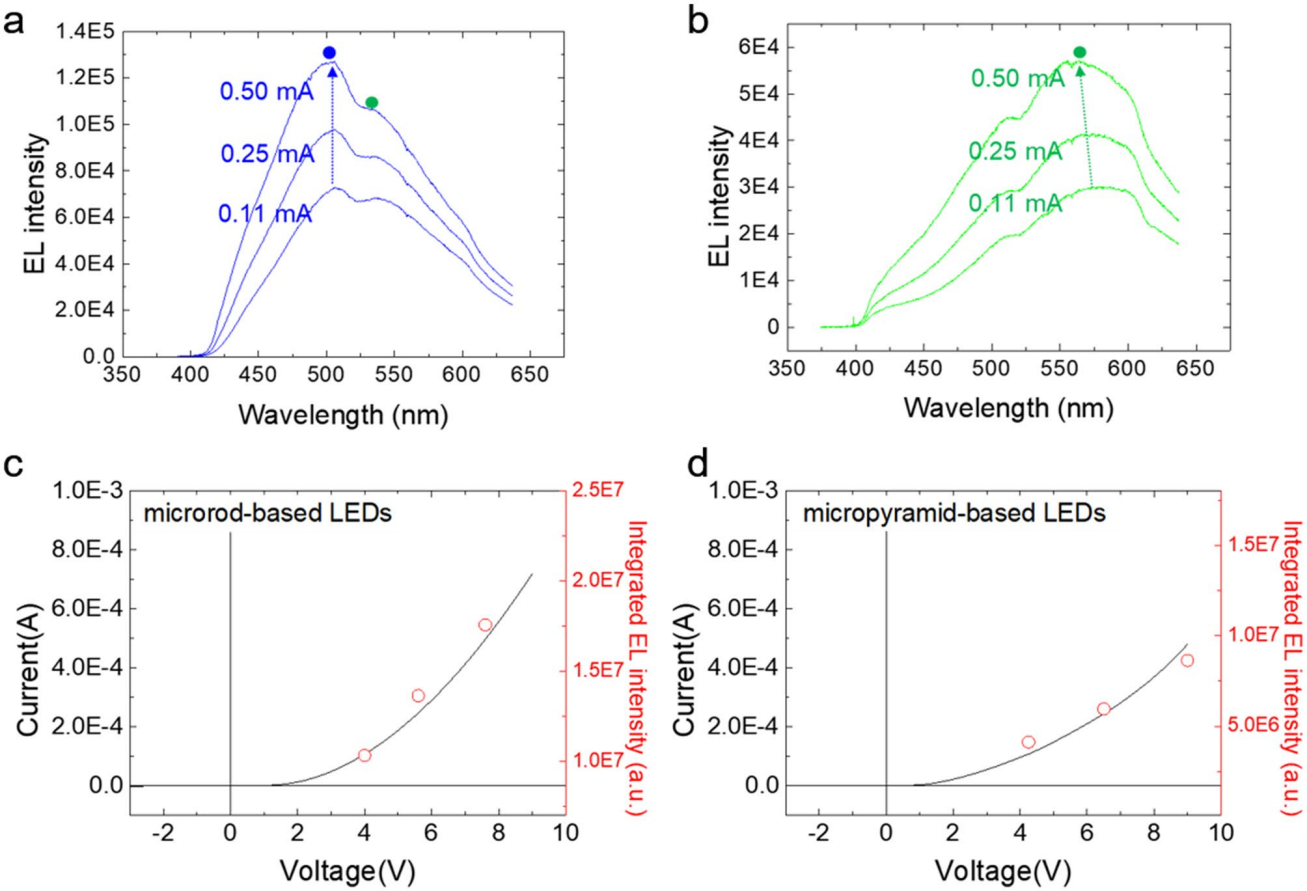
Electroluminescence spectra of (**a**) microrod-based and (**b**) micropyramid-based LEDs for different current levels. *I*–*V* characteristic curves and integrated EL intensity plots of (**c**) microrod-based and (**d**) micropyramid-based LEDs.

The optical characteristics of the microrod- and micropyramid-based LEDs were further investigated by electroluminescence (EL) spectroscopy. The dominant light emission of these LEDs appeared at different wavelengths. For the microrod-based LEDs, the dominant EL emission at an applied current of 0.11 mA was observed at 506.3 nm with a shoulder peak centered at 536.9 nm. As the current level increased to 0.25 and 0.50 mA, the main EL peak shifted to 505.3 and 504.8 nm, respectively. The dominant EL peaks in Fig. 3a were attributed to the QWs formed on the nonpolar planes that predominantly had the microrod structure and emitted blue color at a high current (marked as a blue dot) and the shoulder peak observed at a longer wavelength was attributed to QWs formed on the semipolar planes (marked as a green dot). Meanwhile, Fig. 3b shows that the dominant EL peak of the micropyramid-based LED shifted from 580.3 to 570.2 nm when the applied current was increased from 0.11 to 0.50 mA, with a shoulder peak centered at 509.9 nm. For the micropyramid-based LEDs, the dominant EL peaks were attributed to QWs formed on semipolar planes.

The EL spectra at various current levels also indicated that the shift in the dominant EL peak with increasing current level for the microrod LEDs was much smaller than that for micropyramid LEDs. This behavior presumably resulted from the reduced quantum-confined Stark effect due to the dominant formation of QWs on nonpolar planes for microrod LEDs but on semipolar planes for micropyramid LEDs.

The optical characteristics of the GaN microstructure LEDs can be analyzed by comparing them with previously reported result. Robin et al. reported simultaneous growth of GaN microrod, micropyramid, and microdisk by controlling the diameter of the growth mask opening and fabricated different LEDs by subsequent deposition of InGaN/GaN QWs on those structures^5^. Their observation of photoluminescence (PL) and cathodo-luminescence (CL) shows the similar tendency to our data demonstrating wavelength shift from blue to green and red as the structure changes from microrod to micropyramid and microdisk. In addition, Hong et al. reported the appearance of additional blue emission peak from microrod-based LED at high applied bias voltages with qualitative explanation using the field distribution model^7^. Based on their investigation, EL spectra in Fig. 3a can be interpreted that the QW formed on non-polar plane which dominantly consists the microrod structure emits blue color at a high current (marked as a blue dot), while a shoulder peak at a longer wavelength is observed from the QW formed on semi-polar plane (marked as a green dot). In contrast, the micropyramid-based LED that the semi-polar plane is dominant performs light emissions with a main EL peak at a longer wavelength region whereas a small shoulder peak appeared at a shorter wavelength region (Fig. 3b).

**Figure 4:**
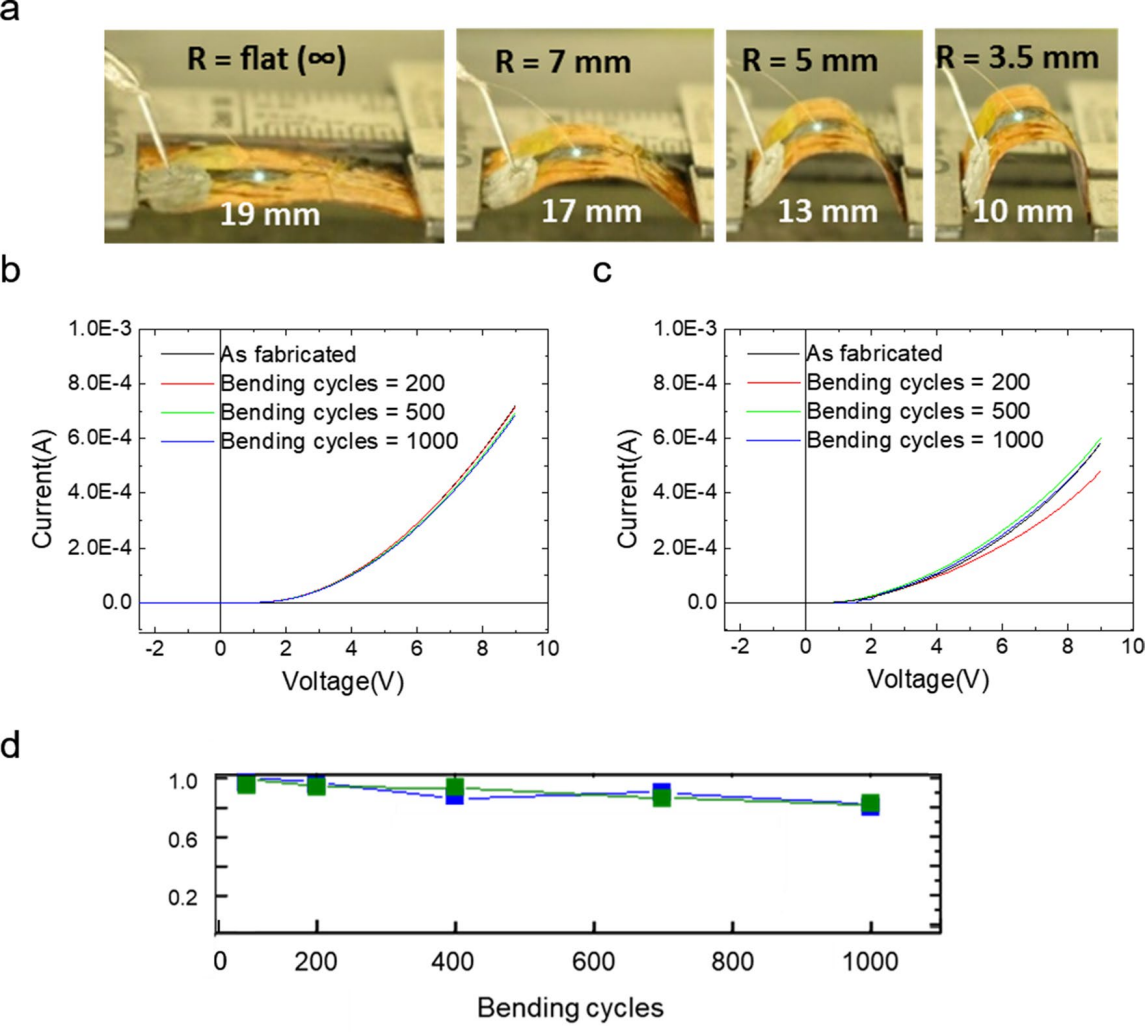
Flexible light-emitting diode (LED) operation. (**a**) Photographs of light emission at bending radii of ∞, 7, 5, and 3.5 mm. Current–voltage characteristic curves after 0 (as-fabricated), 200, 500, and 1000 bending cycles for (**b**) microrod-based and (**c**) micropyramid-based LEDs. (**d**) Normalized electroluminescence intensities of the microrod-based (blue) and micropyramid-based (green) LEDs as a function of bending cycle.

The monolithically integrated multicolor micro-LED arrays fabricated on graphene substrates demonstrated excellent mechanical stability and reliability under severe bending conditions. Figure 4 shows photographs of the light emission at bending radii of ∞, 7, 5, and 3.5 mm and electrical characteristics and normalized EL intensities during 1000 bending cycles. The photographs in Fig. 4a show that when the microrod-based LED array was bent at bending radii of 7, 5, and 3.5 mm, the LEDs reliably emitted blue light. The micropyramid-based LED array also performs stable green light emissions at a bending radii of 3.5 mm (Supplementary Fig. S3).

Figure 4b,c present the current–voltage (*I*–*V*) characteristic curves of the microrod- and micropyramid-based LEDs. The *I*–*V* characteristic curves show typical rectifying behavior LEDs with a turn-on voltage of ≈ 3 V and a leakage current of 3 × 10^−6^ A at –3 V, originated by flows of electrons and holes from *n* to *p* and *p* to *n* layers when a forward bias voltage is applied across the junction resulting in reduction in the potential barrier.

Severe bending did not significantly affect the curves, which exhibited similar rectifying behavior without appreciable differences in the device parameters, such as the turn-on voltage or leakage current.

Figure 4d plots the normalized EL intensities of the microrod- (top) and micropyramid-based LEDs (bottom) as a function of the bending cycle at an applied current of 0.25 mA, where only a small change of less than 20% in the EL intensities was evident after 1000 bending cycles. These results strongly imply that no serious mechanical damage or fracture occurred at the top electrode or at the junctions between the microstructures and graphene during the bending test.

## Conclusion

We fabricated monolithically integrated multicolor LEDs on CVD graphene films for flexible devices, using simultaneous growth of morphology-controlled *n*-GaN microstructures on graphene films. The morphology-controlled growth of the GaN microstructures was realized by optimizing the growth parameters, including the spacing of the ZnO nanotube arrays used as the growth template, and the GaN growth time and temperature. GaN microrod and micropyramid arrays were grown on ZnO nanotubes spaced at 2 and 6 µm at 1000 °C for 10 min. The microrod and micropyramid-based LEDs emitted blue and green light, respectively. Additionally, blue shifting of the EL peaks was observed at higher applied currents from 0.1 to 0.5 mA. The EL peak shift was much smaller for microrod LEDs than for micropyramid LEDs, presumably as a result of the reduced quantum-confined Stark effect. Furthermore, the LEDs fabricated on CVD graphene films were readily transferred onto flexible substrates and exhibited reliable operation during bending. Our study investigates simultaneous growth of non-polar and semi-polar GaN microstructures on CVD graphene films and their fabrications to flexible LEDs to demonstrate multi-color light sources with a minimized blue shifting issue in a flexible form. We believe that the monolithic integrated flexible multicolor LEDs will pave the way for the development of next-generation optoelectronic devices using flexible inorganic LEDs, such as large-scale, high-resolution, and full-color flexible displays.

## Supplementary information

Supplementary Information.
