# Supplementary material for: Flexible and monolithically integrated multicolor light emitting diodes using morphology-controlled GaN microstructures grown on graphene films
Source: Sci Rep. 2020 Nov 12;10:19677. doi: 10.1038/s41598-020-76476-6 (PMC7665051; doi:10.1038/s41598-020-76476-6)
Supplement: Supplementary file 1 — Supplementary Information. [file 41598_2020_76476_MOESM1_ESM.docx]

Supporting Information

**Flexible and monolithically integrated multicolor light emitting diodes using morphology-controlled GaN microstructures grown on graphene films**

*Keundong Lee, Dongha Yoo, Hongseok Oh, and Gyu-Chul Yi*^,^*

Department of Physics and Astronomy, Institute of Applied Physics, and Research Institute of Advanced Materials (RIAM), Seoul National University, Seoul 151-747, Korea

[*]Prof. G.-C. Yi, Department of Physics and Astronomy, Institute of Applied Physics, and Research Institute of Advanced Materials (RIAM), Seoul National University, Seoul 151-747, Korea

^*^E-mail: gcyi@snu.ac.kr


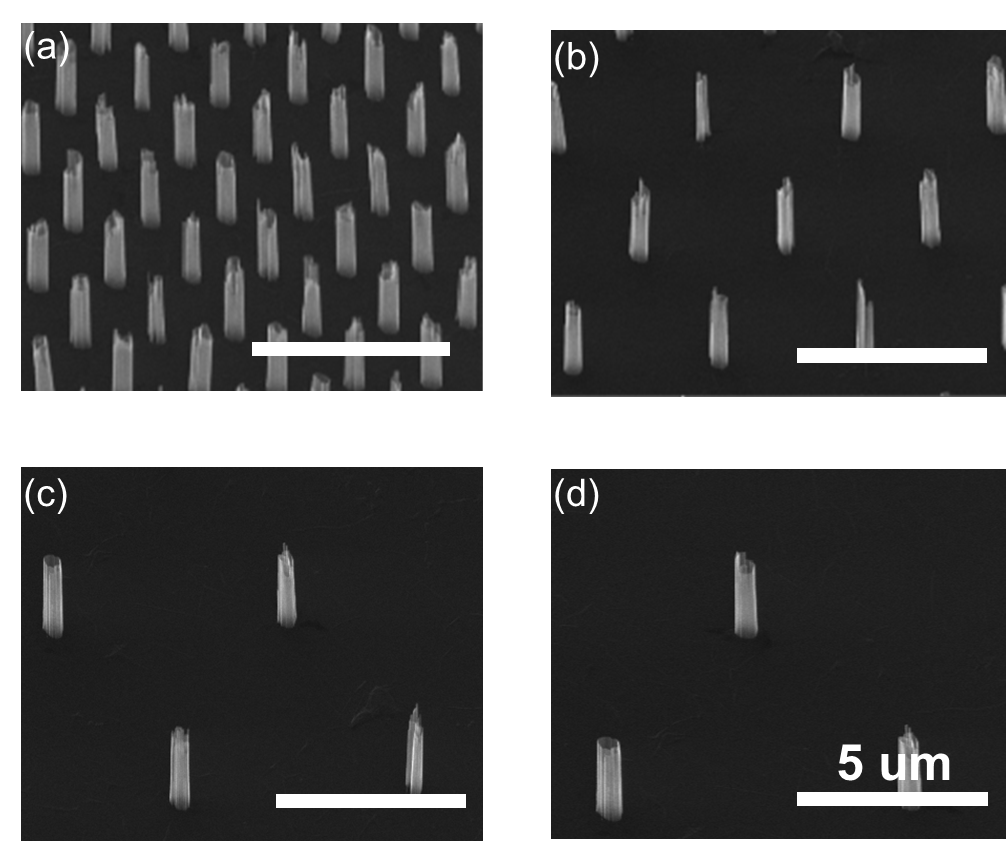


**Figure S1**. Scanning electron microscope images of ZnO nanostructures with different spacings. (a) 2, (b) 4, (c) 6, and (d) 8 μm.

To grow ZnO nanostructures on chemical-vapor-deposited (CVD) graphene films, CVD graphene films were synthesized on metal foils and transferred onto Si substrates with a thin SiO_2_ layer. Then, a thin SiO_2_ film was deposited onto the graphene films as a growth mask. The growth mask for position-controlled growth of nanostructures was formed by varying the lithographic pattern and opening the SiO_2_ growth mask layer. Then, ZnO nanostructures were selectively grown by catalyst-free metal-organic vapor-phase epitaxy (MOVPE). Fig. S1 shows scanning electron microscope images of the ZnO nanostructures with different spacings of 2, 4, 6, and 8 μm and a fixed height and diameter of 3.8 and 0.6 μm, respectively.


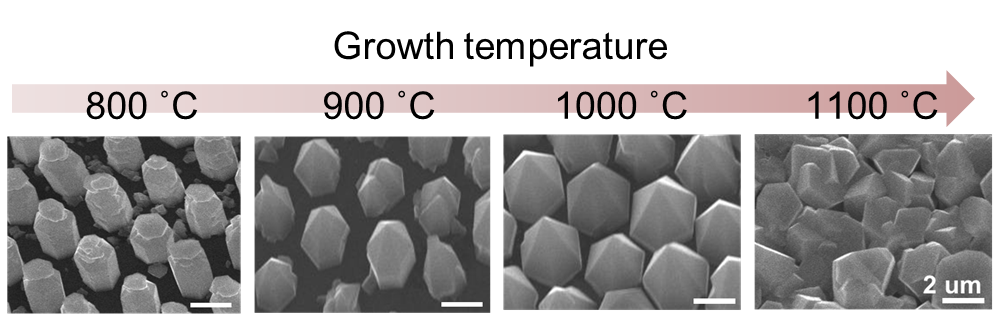


**Figure S2.** Scanning electron microscope images of GaN microstructures grown at various temperatures between 800°C and 1100°C.

We further studied the growth temperature-dependent morphology change in GaN microstructures to investigate the effect of surface diffusion of Ga adatoms. GaN microstructures were grown at various growth temperatures in the range 800–1100°C with a fixed ZnO nanostructure spacing of 4 μm and a length of 3.8 μm (Fig. S2). Increasing the growth temperature changed the morphology from microrod to micropyramid with enhanced growth selectivity. At 1100°C, random GaN particles were observed. These likely resulted from destruction of the ZnO nanostructure due to the high desorption rate of Ga atoms, which resulted in exposure of the ZnO nanostructure to the H_2_ atmosphere at high temperature and dissolution of the ZnO. This result strongly implies that surface diffusion and adsorption/desorption of Ga adatoms play a critical role in the morphology-controlled growth of GaN microstructures.


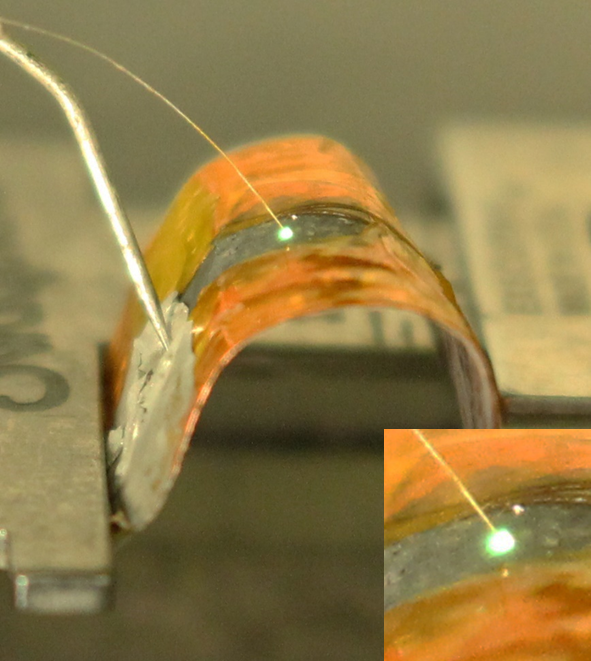


**Figure S3.** Photographs of light emission from micropyramid-based LEDs at a bending radius of 3.5 mm.

Fig. S3 shows that the micropyramid-based LEDs emitted green light at bending radius of 3.5 mm. The materials consisting of the flexible LEDs whose are discrete GaN microstructures and CVD graphene films offer excellent flexibility enabling us to demonstrate stable light emission under severely deformed substrates.
